# Ontogenetic Development of Vestibulo-Ocular Reflexes in Amphibians

**DOI:** 10.3389/fncir.2016.00091

**Published:** 2016-11-08

**Authors:** Francisco Branoner, Boris P. Chagnaud, Hans Straka

**Affiliations:** Department Biology II, Ludwig-Maximilians-University MunichMunich, Germany

**Keywords:** otolith organ, semicircular canal, utricle, extraocular motoneuron, vestibulo-ocular reflex

## Abstract

Vestibulo-ocular reflexes (VOR) ensure gaze stability during locomotion and passively induced head/body movements. In precocial vertebrates such as amphibians, vestibular reflexes are required very early at the onset of locomotor activity. While the formation of inner ears and the assembly of sensory-motor pathways is largely completed soon after hatching, angular and translational/tilt VOR display differential functional onsets and mature with different time courses. Otolith-derived eye movements appear immediately after hatching, whereas the appearance and progressive amelioration of semicircular canal-evoked eye movements is delayed and dependent on the acquisition of sufficiently large semicircular canal diameters. Moreover, semicircular canal functionality is also required to tune the initially omnidirectional otolith-derived VOR. The tuning is due to a reinforcement of those vestibulo-ocular connections that are co-activated by semicircular canal and otolith inputs during natural head/body motion. This suggests that molecular mechanisms initially guide the basic ontogenetic wiring, whereas semicircular canal-dependent activity is required to establish the spatio-temporal specificity of the reflex. While a robust VOR is activated during passive head/body movements, locomotor efference copies provide the major source for compensatory eye movements during tail- and limb-based swimming of larval and adult frogs. The integration of active/passive motion-related signals for gaze stabilization occurs in central vestibular neurons that are arranged as segmentally iterated functional groups along rhombomere 1–8. However, at variance with the topographic maps of most other sensory systems, the sensory-motor transformation of motion-related signals occurs in segmentally specific neuronal groups defined by the extraocular motor output targets.

## Introduction

Spatially accurate perception of the visual world in all vertebrates requires sufficiently long periods of gaze stability given the relatively slow processing of images in the widespread visual circuits (Straka and Dieringer, 2004; Angelaki and Hess, 2005; Angelaki and Cullen, 2008). This requirement is particularly challenged during locomotion when concurrent 3d-head/body movements cause considerable retinal image displacements. Without an image stabilizing mechanism, motion-induced changes of relative eye position would cause blurred vision due to retinal image slip and impaired visual signal processing. Therefore, counteracting eye/head movements during self- (and passive) motion compensate image motion and thereby ensure visual acuity. This reaction has been classically allocated to the sensory-motor transformation of motion-related feedback signals from several sense organs, which encode motion-related vestibular, visual and proprioceptive signals (Straka and Dieringer, 2004; Straka et al., 2014). Image stabilization thus results from conjointly acting multi-sensory reflexes, each with a specific contribution over the dynamic range of head/body motion (Angelaki and Cullen, 2008).

While the classical description of a sensory-driven mechanism for gaze stabilization represents the generally accepted view, physiological evidences in primates, fishes and amphibians have challenged the exclusive role of these signals for gaze control during active head movements and locomotor activity (Harris, 1965; Cullen, 2004; Chagnaud et al., 2012; Lambert et al., 2012). Efference copies of neck motor commands during voluntary head movements in primates, for instance, influence central vestibular signal processing (Cullen, 2004, 2011). Furthermore, spinal locomotor efference copies during swimming in *Xenopus* tadpoles (Combes et al., 2008; Lambert et al., 2012; von Uckermann et al., 2016) and adults (von Uckermann et al., 2013) directly access the extraocular motor nuclei and trigger spatio-temporally adequate compensatory eye movements. At the same time these efference copies attenuate peripheral vestibular signal encoding (Chagnaud et al., 2015). Accordingly, maintaining stable visual images during locomotion depends on both, intrinsic signals related to the self-motion as well as motion-related sensory feedback.

The predominating vertebrate motion detection system is the vestibular system with semicircular canals and otolith organs in the inner ear as motion-sensitive sensory structures. The embryonic formation and post-embryonic maturation of endorgans and neural circuitries for sensory-motor transformations are indispensable requirements for self-motion and encoding of motion-associated head movements. An early functional onset of vestibular reflexes during ontogeny is therefore one of the prerequisites for all precoccial vertebrates such as most amphibians with a self-sustaining lifestyle. The early onset of locomotor activity in amphibians (e.g., *Xenopus*: Kahn et al., 1982) suggests that vestibular circuitries are sufficiently well established at that time. Although there is no direct experimental evidence yet for optokinetically driven eye movements in tadpoles smaller than stage 45 (Lambert et al., 2008), spontaneous eye twitches occur at locomotor onset. This suggests that at this developmental period all neuronal elements of the vestibulo-ocular reflexes (VOR) circuitry are interconnected and capable to detect body movements and to trigger motor reactions. However, the different mechanistic principles for motion detection by semicircular canals and otolith organs suggest that the respective VOR behaviors potentially have different functional onsets and maturation time courses.

Deciphering the ontogenetic establishment of vestibular reflexes poses particular requirements to the experimental paradigm. Most important is the accessibility of experimental animals at the developmental stage of behavior onset as well as the possibility to apply methods to quantify the respective behavior and to electrophysiologically probe the neuronal circuitry. Amphibians are excellently suited for such studies given the availability of embryonic and larval stages. Moreover, the early onset of locomotor activity and the possibility to employ isolated preparations facilitates the quantification of motor behavior and network activity (Straka and Simmers, 2012). Accordingly, studies on larval frogs revealed the critical parameters that determine the onset and maturation of angular and linear VOR behavior as well as the ontogenetic origin of the underlying neuronal elements. Moreover, the activation of fictive locomotion in isolated tadpole preparations allowed specifying the contribution of motor efference copies to gaze stabilization.

## Morphological and Neuronal Substrates for Vestibulo-Ocular Reflexes

**Figure 1:**
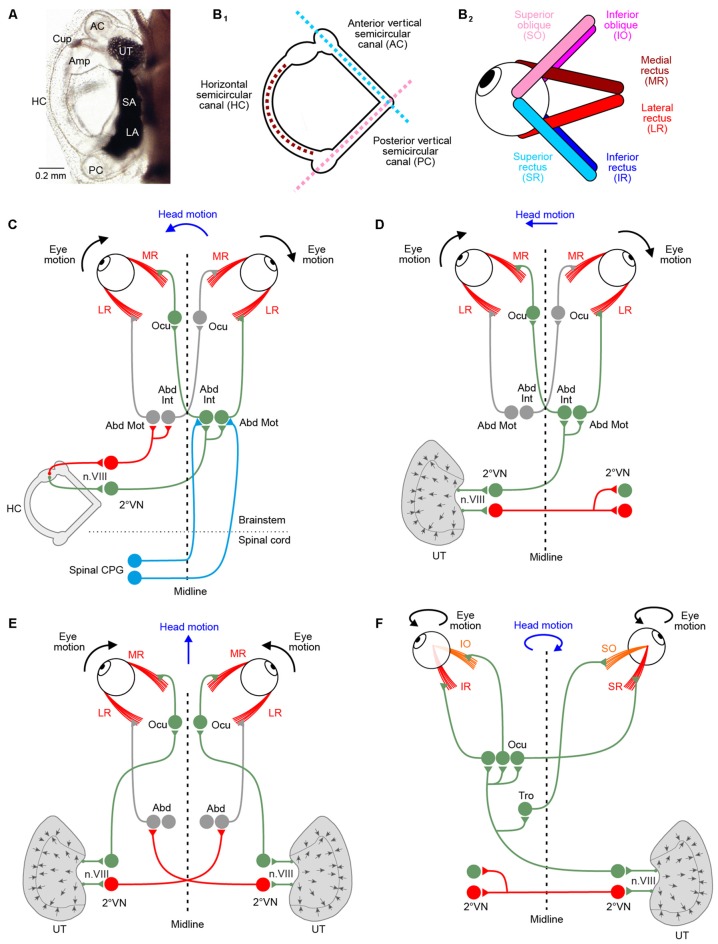
**Morphological substrate underlying angular and linear vestibulo-ocular reflexes (VOR). (A)** Dorsal view of the left otic capsule of a tadpole at developmental stage 49, depicting the location and arrangement of all vestibular endorgans. **(B)** Schematics depicting the spatial arrangement of the three semicircular canals of the left labyrinth **(**B_1_**)** and the six extraocular muscles of the left eye **(B_2_**); note that color-coded antagonistic pairs of eye muscles are spatially aligned with individual semicircular canals. **(C–F)** Schematics depicting the three-neuronal pathways between vestibular endorgans and eye muscles for mediating horizontal angular **(C)**, horizontal linear **(D,E)** and vertical/oblique tilt **(F)** VOR; note the activation of conjugated eye movements during left-right **(D)** and vergence eye movements during forward-backward **(E)** translational head motion; during undulatory tail-based swimming in tadpoles, ascending spinal pathways (light blue connections in **C**) mediate locomotor efference copies directly onto abducens motoneurons (Abd Mot) and internuclear neurons (Abd Int). Green, red projections indicate excitatory and inhibitory connections, respectively. 2°VN, second-order vestibular neurons; Abd, abducens nucleus; AC, anterior vertical semicircular canal; Amp, ampulla; Cup, cupula; HC, horizontal semicircular canal; LA, lagena; Ocu, oculomotor nucleus; PC, posterior vertical canal; SA, saccule; Tro, trochlear nucleus; UT, utricle.

Naturally occurring head movements are detected in all vertebrates by evolutionarily conserved vestibular sense organs in the inner ear (Fritzsch and Straka, 2014). Head movements generally contain angular and linear acceleration components, which are decomposed by two different types of vestibular organs. As exemplarily shown for *Xenopus laevis* tadpoles in Figure 1A, vestibular organs subdivide into three perpendicularly oriented semicircular canals (anterior vertical, posterior vertical and horizontal) and three otolith organs (utricle, saccule, lagena). This pattern is present in all jawed vertebrates, except therian mammals, which lack a lagena (de Burlet, 1929). Angular acceleration is detected by semicircular canals and depends on the inertia of the endolymph within the tubular structure. Linear translation is detected by otolith organs and depends on the inertia of a heavy mass relative to the epithelial sensor. The cellular receptors for motion detection are ciliated mechanosensory hair cells embedded in the epithelia of the different organs (Hudspeth, 2005). Each hair cell has multiple cilia at the apical side with a staircase arrangement that assigns a direction-specific functional polarity to the cilial bundle. Shearing of the bundle towards/away from the kinocilium causes a depolarization/hyperpolarization of the hair cell, respectively (Hudspeth, 2005). While all hair cells in a given semicircular canal have the same directional sensitivity, the preferential vector orientation of otolith hair cells extends over 360° in each of the latter organs (Wersäll, 1956).

Following transduction of motion stimuli, alterations of the hair cell membrane potential are synaptically transmitted onto vestibular afferent fibers and encoded as modulated spike discharge (Goldberg, 2000). These signals are relayed to the central vestibular neurons in the hindbrain *via* parallel signaling pathways with respect to endorgan origin and response dynamics (Straka and Dieringer, 2004; Straka et al., 2009). Central vestibular neurons form the key element of the sensory-motor transformation underlying VOR (Straka et al., 2005). However, the processing of afferent signals from the different semicircular canal and otolith organs largely occurs in separate groups of vestibular neurons (Straka et al., 2002b). Depending on the spatial origin of the afferent input, the motion-induced spike discharge is then distributed to motoneurons in the oculomotor, trochlear and abducens nuclei (Straka et al., 2002a).

In amphibians as in all other vertebrates, motoneurons in the three ocular motor nuclei innervate six eye muscles that generate the three-dimensional VOR. The muscles are innervated by sets of extraocular motoneurons: medial rectus (MR), superior rectus (SR), inferior rectus (IR) and inferior oblique (IO) motoneurons are located in the oculomotor nucleus, superior oblique (SO) motoneurons in the trochlear and lateral rectus (LR) motoneurons in the abducens nucleus (Straka et al., 2014). The six extraocular muscles are attached to each eye in an evolutionarily conserved topographic pattern (Figure 1B_2_; Simpson and Graf, 1981; Fritzsch et al., 1990). The absence of a bony orbit in amphibians, particularly in the larvae, facilitates the identification of individual muscles and corresponding extraocular motor nerve insertions and allows determining the respective pulling directions (Straka and Simmers, 2012). In contrast to birds or mammals, amphibian eye muscles insert directly at the eyeball and the cartilaginous or bony skeletal elements without tendonous structures. Eye muscles thereby form three antagonistic pairs, based on pulling directions: LR—MR muscle; SO—IO muscle; SR—IR muscle (Figure 1B_2_). Interestingly, the pulling directions of the three muscle pairs are approximately aligned with the orientation of the semicircular canal planes (see matching color-code in Figures 1B_1,2_).

The pathway from any vestibular endorgan to an extraocular muscle comprises three consecutive neurons: vestibular nerve afferents, second-order vestibular neurons (2°VN) and extraocular motoneurons (Straka and Dieringer, 2004). This three-neuronal circuitry was already postulated very early, despite the lack of high-resolution morpho-physiological methods (Lorente de Nó, 1933; Szentágothai, 1943). The functional organization of this network has been highly conserved during evolution from early vertebrates to mammals (Figures 1C–F; Straka and Baker, 2013; Straka et al., 2014). Moreover, the relative simplicity of this pathway triggered numerous investigations of the underlying computational processes and made it a uniquely suited system for studying sensory-motor transformation in general (see Robinson, 1985).

## Behavioral Onset of Vestibular Reflexes

**Figure 2:**
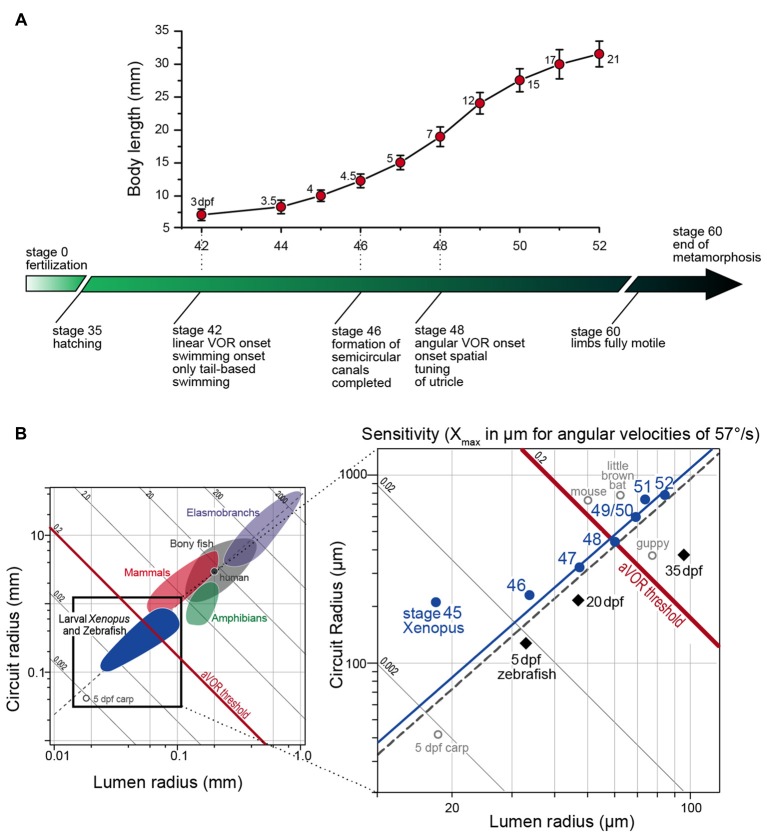
**Progression of larval body growth and major steps in vestibular reflex development in *Xenopus laevis*. (A)** Dependency of body length (mean ± SD) on developmental stage and onset/maturation of critical aspects of vestibulo-ocular response behavior (adapted from Lambert et al., 2008). Numbers next to each marker on the graph indicate the age of the larvae in days post-fertilization (dpf). **(B)** Plot of mean canal lumen radius vs. mean canal circuit radius of the horizontal canals of larval *Xenopus* and zebrafish with superimposed canal dimensions for selected classes of vertebrates (left in **B**) adapted from Muller (1999) and Lambert et al. (2008). Oblique lines represent theoretical endolymph displacement in μm for a given angular velocity (1 rad/s or 57.3°/s) and the dashed gray line is the regression line of *R* = 38.9 × *r*^1.60^ (see Muller, 1999). The solid blue line is the regression line of *R* = 42.2 × *r*^1.59^ for stage 47–52 *Xenopus* larvae.

Natural head/body movements consist of dynamically altering combinations of angular and linear acceleration components, which are detected and decomposed into individual vector components by semicircular canal and otolith organs. Based on their different motion detection mechanisms, the functional onset and maturation of the directional tuning might have different constraints. For example in *Xenopus* larvae, the known progression of body and inner ear growth during ontogeny provides the temporal framework onto which the onset and various aspects of the functional maturation of vestibular-evoked eye movements can be placed (see lower time axis in Figure 2A).

### Semicircular Canal Reflexes

The behavioral onset of the semicircular canal-related angular VOR in *Xenopus laevis* tadpoles was determined during passive rotation at different developmental stages (Lambert et al., 2008). Respective experiments indicated that the horizontal angular VOR becomes functional around stage 48, which corresponds to a time when tadpoles already swim freely for several days. The obvious absence of a horizontal angular VOR during early larval locomotion is surprising, given the necessity of compensatory eye movements for retinal image stabilization. The relatively late onset of the angular VOR, however, is not related to extraocular motor deficits since otolith- or optokinetically-driven eye movements can be elicited as early as stage 42 (Horn et al., 1986; Lambert et al., 2008), which coincides with the onset of longer swimming episodes (Currie et al., 2016). This suggests that all structural elements, at least for visual and otolith-driven reflexes, are functionally intact and appropriately interconnected. Given the convergence of otolith and semicircular canal signals in 2°VN (Straka et al., 2002b), the failure to transmit signals from the latter vestibular endorgans must derive from a functional incapacity of the semicircular canal sensory periphery. The integrity of the circuitry underlying horizontal semicircular canal (HC)-derived reflexes was tested by electrical semicircular canal nerve stimulation and pressure pulse-induced endolymph displacements of the cupula at stage 47, before the onset of the horizontal angular VOR. These experiments confirmed the full operational capacity of extraocular muscles and central neural pathways, including appropriate response latencies and dynamics. However, more importantly, the experiments also demonstrated the ability of semicircular canal hair cells to evoke graded responses. Thus, the absence of a horizontal angular VOR prior to stage 48 is not related to a general failure of hair cells to sense endolymph motion or to transmit the transduced neural signals onto vestibular afferents. It rather suggests that a passive element of the HC is the limiting factor for the ontogenetic onset of the reflex.

The capacity to detect angular acceleration during head rotation through cupula displacements is critically influenced by the inertia of the endolymph within the tubular structure. This, in turn is directly related to the diameter and the length of the semicircular canals. Accordingly, the endolymph movement, required to sufficiently displace the cupula and shear hair cell cilial bundles, depends on the canal lumen and circuit radius (Oman et al., 1987; Muller, 1999). The resistance for fluid motion through a tube is inversely proportional to the fourth power of the tube radius (Hagen-Poiseuille law). Thus, this parameter is highly relevant for the capability of the semicircular canals to detect angular head motion. Accordingly, the physical dimensions of the semicircular canals are the likely determinant for the functional onset of the angular VOR. Comparison of HC lumen and circuit radii before and after the functional onset of the angular VOR indicated that first reflex measurements coincided with a significant increase in canal dimensions between stages 47 and 48 (Lambert et al., 2008). The earliest above-threshold discharge modulation of the LR nerve was encountered at stage 48, when the average canal lumen and circuit radii were ~60 μm and ~0.44 mm, respectively (right in Figure 2B). These values comply well with theoretically predicted duct dimensions necessary for an undisturbed laminar endolymph flow (Muller, 1999).

The hypothesis that semicircular canal size determines the functional onset of the angular VOR was verified by comparing vertical semicircular canal dimensions and the onset of the vertical angular VOR in *Xenopus* tadpoles (Lambert et al., 2008). In fact, a modulated SO nerve activity occurred in stage 47 but not in stage 46 tadpoles, despite anatomically complete semicircular canals and fully functional VOR circuitry including extraocular motoneurons and eye muscles (Lambert et al., 2008). The lumen radii of the vertical semicircular canals at the onset of the vertical angular VOR were also ~60 μm as found for the HC at the onset of the horizontal angular VOR. Thus, independent of the developmental stage all three semicircular canals had similar lumen radii at the functional onset of the respective angular VOR (Lambert et al., 2008). This intrinsic comparison thus supports the hypothesis that semicircular canal size determines the angular VOR onset during *Xenopus* ontogeny, a conclusion that was confirmed in larval zebrafish (Figure 2B; Beck et al., 2004). This further suggests that very small vertebrates, such as amphibian or fish larvae (see left plot in Figure 2B) must grow rapidly to acquire sufficiently sized semicircular canals that allow the detection of angular acceleration (Muller, 1999).

The conclusion that semicircular canals in *Xenopus* tadpoles need to acquire a particular size to detect angular acceleration is, however, based on a test stimulus acceleration magnitude of 400°/s^2^ (Lambert et al., 2008). This is a critical assumption, since the absence of a horizontal angular VOR in larval *Xenopus* at stage 46–48 depends on the fact that naturally occurring accelerations do not exceed this value. This, however, has been recently challenged by a study of ontogenetic changes in free swimming *Xenopus* tadpoles, demonstrating that these animals reach angular accelerations of up to 10,000°/s^2^ during self-motion (Hänzi et al., 2015b). This is considerably higher than the value used to determine the developmental onset of the VOR in the previous study. This suggests that semicircular canals would be in fact capable of detecting naturally occurring angular accelerations during locomotor activity at much younger stages. It is therefore quite possible that semicircular canals become already functional for detecting self-motion immediately after structural completion at stage 46 (Haddon and Lewis, 1991; Bever et al., 2003; Quick and Serrano, 2005). Therefore, high stimulus acceleration magnitudes such as those generated during swimming could theoretically elicit an angular VOR. This interpretation, however, requires closer investigation, since vestibular signal processing during rhythmic swimming is considerably attenuated (Chagnaud et al., 2015). This complies with the suppression of the horizontal angular VOR by ascending spinal locomotor efference copies during actively induced body motion (Lambert et al., 2012). Thus, even though swimming-related angular accelerations could be theoretically detected by very young larvae, peripheral sensory attenuation and central cancellation during self-motion likely prevents an activation of an angular VOR. However, during externally induced passive perturbations of the head/body with lower acceleration profiles, semicircular canal size plays the dominant role in determining the effective sensitivity. This would explain the delayed functional onset of the VOR with respect to completion of the tubular structure as previously suggested (Lambert et al., 2008).

### Functional Tuning of Otolith Reflexes

Otolith-dependent counter-rotations of the eyes have been observed in *Xenopus* tadpoles early after hatching at stage 42 (Horn et al., 1986; Lambert et al., 2008). However, the early appearance of roll-induced eye movements does not necessarily imply that the underlying extraocular motor commands are spatially specific. In fact, directionally appropriate compensatory eye movements require that spatially specific signals from particular regions of the utricular epithelium are mediated onto extraocular motoneurons with matching directional selectivity. The 360° horizontal directionality of the utricular hair cell epithelium, however, challenges a simple vector-oriented establishment of VOR connections between specific utricular sectors and sets of extraocular muscles (Rohregger and Dieringer, 2002). One possibility is a wiring mechanism that depends on genetically encoded molecular markers that allow utricular hair cells with specific directional preferences to be linked with respective vestibular afferent fibers; these in turn would then connect to corresponding sets of 2°VNs that project to specific sets of extraocular motoneurons and eye muscles. While this scenario is not entirely unlikely, it is difficult to imagine that such a hard-wired solution would provide a sufficiently precise spatial specificity for connections between utricular sectors and extraocular motoneurons (Rohregger and Dieringer, 2002). Alternatively, the establishment of spatially appropriate wiring between sectors of utricular hair cells and sets of eye muscles could be spatially fine-tuned along a reference frame such as the visual motion detection system in the pretectum or the orthogonal semicircular canal system (Figures 1B_1,2_; Simpson and Graf, 1981).

**Figure 3:**
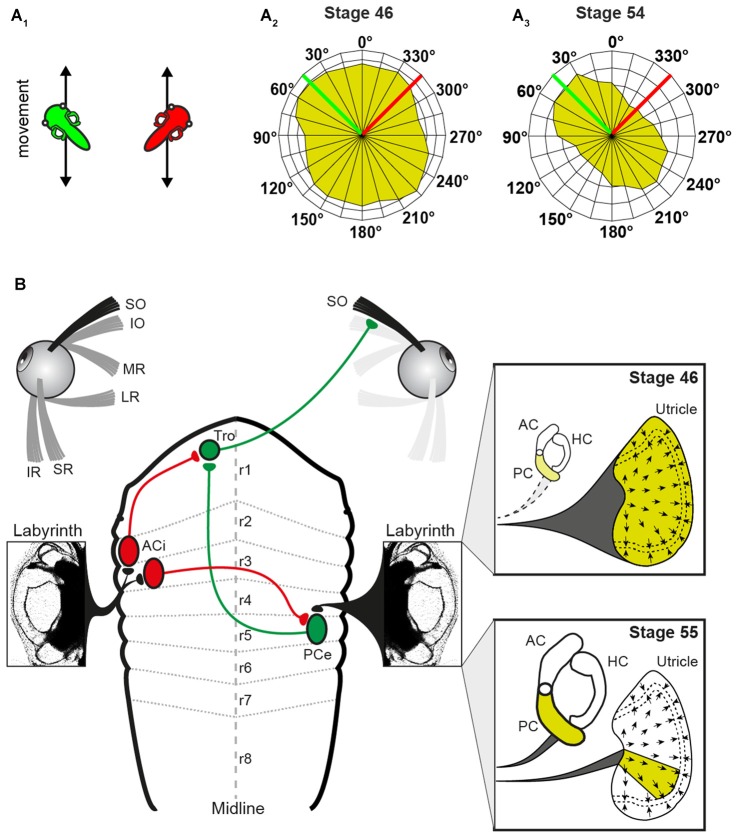
**Schematic, depicting altered peripheral sensory contributions during the maturation of the linear VOR. (A)** Scheme, illustrating 2 out of 24 translational motion directions **(A_1_)** used to quantify the superior oblique (SO) nerve tuning (tadpoles indicate a linear translation along (green) and orthogonally (red) to the SO muscle/ipsilateral PC; SO response vector plot of all 24 motion directions in a stage 46 **(A_2_**) and a stage 55 tadpole **(A_3_**) green and red lines indicate the directions of the linear translations shown in **(A_1_**). **(B)** Circuitry depicting the connections between the utricle/PC and the SO muscle. Note that SO nerve responses derive from the entire utricle (yellow area in upper inset) at stage 46 and from a particular utricular sector that aligns with the PC at stage 55 (yellow area in lower inset). AC, anterior vertical semicircular canal; ACi, inhibitory AC neuron; HC, horizontal semicircular canal; IO, inferior oblique; IR, inferior rectus; LR, lateral rectus; MR, medial rectus; PCe, excitatory PC neuron; r1–8, rhombomere 1–8; SR, superior rectus; Tro, trochlear nucleus. Adapted from Branoner and Straka (2015).

Roll motion in *Xenopus* larvae elicits eye movements already at stage 42 (Horn et al., 1986). Experiments, in which the SO nerve was recorded in *Xenopus* tadpoles during linear translation in different directions in the horizontal plane (for two orthogonal directions see Figure 3A_1_) allowed determining the utricular tuning of this extraocular motor nerve (Branoner and Straka, 2015). At stage 46–48, SO motoneurons receive omnidirectional utricular signals during horizontal translational motion (Figure 3A_2_), indicating an absence of spatial tuning (Branoner and Straka, 2015). Between stage 49 and 53, SO motoneurons receive otolith inputs with a gradually increasing spatial tuning. At the end of this period, the directional selectivity of the utricular signal, mediated onto SO motoneurons, is narrowed (Figure 3A_3_). In fact, the utricular sector (i.e., the optimal hair cell polarization) from which the SO nerve responses originate is now aligned with the ipsilateral posterior vertical semicircular canal (PC) plane (Figure 3B). This alignment suggests that the latter endorgan might provide the reference frame after its functional onset and assist in the directional tuning of otolith responses. This hypothesis was confirmed by selectively suppressing the formation of bilateral semicircular canals by bilateral injections of hyaluronidase into the otic capsule in *Xenopus* tadpoles at stage 44, i.e., before the anatomical completion of these endorgans (Haddon and Lewis, 1991). At variance with the absence of an angular VOR in these semicircular canal deficient (hyaluronidase-treated) *Xenopus* tadpoles at stage 52 (Branoner and Straka, 2015), cyclic sinusoidal translation caused a modulation of the SO nerve activity, indicating a functionally intact utricle. However, in the absence of semicircular canals, the modulated SO nerve activity of tadpoles at stage 53 had no preferential translational vector orientation and thus remained omnidirectional and very similar to that of young *Xenopus* tadpoles at stage 47 (Branoner and Straka, 2015). Accordingly, the lack of directionality of otolith-derived extraocular motor responses in contrast to those of age-matched controls demonstrates a lack of developmental tuning of otolith inputs in SO motoneurons in the absence of semicircular canals. This suggests that the maturation of otolith response directionality during translational motion in extraocular motoneurons depends on functional semicircular canals. The mechanism of this maturation process likely includes stabilization of co-activated sensory inputs from a semicircular canal and a utricular epithelial sector with spatially aligned hair cell specificity during e.g., roll/tilt head/body motion. This might occur either at the level of 2°VN or further downstream at the level of SO motoneurons (Figure 3B).

## Locomotor Efference Copy-Driven Eye Movements

Gaze stabilization in vertebrates has been classically assigned to the transformation of motion-related sensory signals into extraocular motor commands (Angelaki and Cullen, 2008). However, several studies in larval and adult *Xenopus* have revealed a major role of locomotor efference copies in retinal image stabilization during tail and limb-based swimming (Combes et al., 2008; Lambert et al., 2012; von Uckermann et al., 2013, 2016). In fact, copies of the central pattern generator (CPG)-derived locomotor output directly trigger conjugate eye movements in larval *Xenopus* (Combes et al., 2008; Lambert et al., 2012). These eye movements counteract oppositely directed horizontal head displacements during undulatory tail-based locomotion in the intact animal. The underlying synchronous extraocular motor burst activity of LR and synergistic MR motoneurons on the opposite side is strictly coupled with each spinal ventral root burst. The efference copy signals that provoke these rhythmic extraocular bursts involve neurons in the dorsolateral cord region of the first 10 spinal segments and are transmitted by direct ascending pathways to contralateral LR motoneurons (Figure 1C). In addition, re-crossing abducens internuclear neurons to MR motoneurons on the opposite side support conjugated eye movements (Figure 1C). The spino-extraocular motor coupling is spatially specific for the horizontal plane and occurs only during fast locomotion that causes substantial left-right head displacements during undulatory axial swimming (Lambert et al., 2012). While the presence of this mechanism has been well established at mid-larval stages (stage 55), it is very likely that the spino-extraocular motor coupling is already functional as soon as larvae start swimming at stage 42. This suggests that locomotor efference copies potentially represent the major if not exclusive source for gaze stabilization during self-motion in these animals.

The employment of locomotor efference copies in larvae might be a particularity due to the relatively simple locomotor strategy of undulatory swimming. However, towards the end of larval development, the animals start altering the locomotor style and gradually switch from tail-based undulatory movements to bilaterally synchronous hindlimb kicking (Combes et al., 2004). In fact, over several developmental stages at metamorphic climax both locomotor types occur simultaneously (Combes et al., 2004; von Uckermann et al., 2016). This change in locomotor strategy results in head/body movements with altered dynamics and visual consequences. This switch necessitates a concurrent alteration in compensatory eye movements from conjugate left-right rotations in larvae to convergent movements during rhythmic linear forward acceleration produced by each kick cycle in the adult frog. In fact, forward propulsion that is produced by synchronous burst activity in bilateral hindlimb extensor muscles is accompanied in isolated *Xenopus* preparations by concurrent bursts in MR motoneurons of both eyes (von Uckermann et al., 2013). This extraocular discharge pattern produces a cyclic convergence of both eyes in the intact animal as predicted from the head motion pattern during limb-based swimming in the adult frog. The transition in propulsive strategy is a gradual process with adaptive interactions between locomotor and extraocular motor circuitry. This allows a constant match of spatio-temporal requirements for retinal image stabilization when one mechanism emerges and replaces another (von Uckermann et al., 2016). At metamorphic climax, spino-extraocular motor coupling originates from both, the hindlimb and the axial CPGs, with the latter’s influence becoming gradually weaker and finally ceasing at the end of metamorphosis when the tail is resorbed (von Uckermann et al., 2016).

Spinal locomotor efference copies might provide the sole mechanism to produce conjugate left-right eye movements and thus offset the visual disturbances during swimming in young larvae, before HC formation (Lambert et al., 2012). Compatible with these observations, the intrinsic signals remain the predominant if not exclusive source for gaze stabilization during locomotion in older larvae. Although the impact of the CPG efference copy-driven motor commands perfectly matches the spatio-temporal eye motion pattern of the horizontal angular VOR, the two different signals do not contribute jointly to produce a common signal for image stabilization during swimming (Lambert et al., 2012). In fact, when the spinal CPG is active, the horizontal angular VOR, resulting from concurrent passive head movements is suppressed. This indicates that even though semicircular canals are structurally complete at stage 46 and potentially capable of sensing the high acceleration that occurs during self-generated undulatory swimming (Hänzi et al., 2015b), gaze stabilizing eye movements derive exclusively from intrinsic locomotor efference copies. The suppression of the horizontal angular VOR in larval *Xenopus* is in part accomplished at the level of the sensory periphery (Chagnaud et al., 2015). Efferent neurons that project to vestibular hair cells display cyclic impulse bursts during swimming that directly derive from the spinal CPG. These locomotor efference copies cause an overall gain reduction of the signal encoding in vestibular afferent fibers by more than 40%, likely preventing saturation of the sensory signal processing in central circuits. Thus, the vestibular periphery’s sensitivity is adjusted to the altered stimulus statistics during locomotion. Whether an additional attenuation of vestibular sensory signals occurs at the level of 2°VN during amphibian locomotion, as shown for primate central vestibular neurons during active head movements (Cullen, 2004, 2011), remains to be shown.

The finding that locomotor efference copy-triggered eye movements are the predominating mechanism for gaze stabilization challenges our traditional view of how animals offset the disruptive effects of propulsive body movements on visual processing. The cancellation of externally induced motion signals during locomotion shows that efference copies of locomotor-related rhythmic neural signals supplant, rather than supplement the VOR. The presence of such a mechanism not only in larval amphibians with a fish-like swimming pattern, but also in adult frogs with a limb-based locomotor style suggests that gaze stabilization through predictive intrinsic signals can be generalized. As evidenced from studies on dogs and human patients, a similar mechanism is likely also implemented in vertebrates with more complex locomotor dynamics (Brandt et al., 1999, 2001). This suggests that efference copies play an important if not exclusive role for gaze stabilization during highly predictable rhythmic locomotor activities, while sensory-motor transformations, in particular of vestibular signals, stabilize retinal images during unpredictable consequences of passive motion.

## Functional Organization of Vestibular Circuitries

The functional onset and maturation of the linear and angular VOR is preceded during ontogeny by the formation of sensory-motor components and the establishment of appropriate neuronal circuitries. In particular, the spatio-temporal specificity of the VOR benefits from a precise wiring and specific topographical arrangement of all involved neuronal elements.

### Ontogeny of the Sensory Periphery and Vestibular Nerve Afferent Projections

In all vertebrates including amphibians, semicircular canal and otolith organs in the inner ear develop from placodal regions of the ectoderm at the level of hindbrain segment 5 and 6 (Fritzsch and Straka, 2014). Asymmetric cell proliferation processes cause an invagination of the epithelial region into an otocyst within which distinct cellular patches transform into several vestibular endorgans (Fritzsch and Straka, 2014). The formation of sensory epithelia, primary afferent neurons and functionally appropriate connections between the two structural elements are genetically regulated by paraxial mesodermal as well as ectodermal tissue (Fritzsch et al., 2002, 2007; Fritzsch, 2003). The similarity of the underlying cellular and molecular processes between *Xenopus* (Quick and Serrano, 2007) and other vertebrates complies with a common regulatory scheme of inner ear formation and endorgan differentiation (Straka, 2010). This is mostly due to the combinatorial expression of evolutionary conserved genetic regulatory elements (Fritzsch, 2003). Vestibular endorgans derive from specific otic placodal areas that are controlled by regionally different genetic patterns (Kil and Collazo, 2001). However, the timing of formation and maturation differs for semicircular canal and otolith organs as shown in *Xenopus* (Nieuwkoop and Faber, 1994; Quick and Serrano, 2005). While the utricular macula is already well developed at larval stage 42/43 (Nieuwkoop and Faber, 1994), semicircular canals appear initially as paired axial protrusions early after hatching (stage 43). During subsequent development these protrusions fuse and finally become morphologically complete at stage 46–47 (Haddon and Lewis, 1991) at ~5 days post-fertilization (Bever et al., 2003). A differential timing of semicircular canal and otolith organ formation has also been observed in salamanders (Wiederhold et al., 1995) and zebrafish (Haddon and Lewis, 1996; Bever and Fekete, 2002; Whitfield et al., 2002). Thus, a relatively late morphological completion of semicircular canals likely represents a general feature of vestibular organ development in vertebrates. This however might be related to the different anatomical complexity of the two types of vestibular endorgans (Straka et al., 2014). In fact, otolith organs, which also precede the evolutionary appearance of semicircular canals (Fritzsch and Straka, 2014), are structurally relatively simpler organs compared to the more specialized and derived tubular structure.

Vestibular nerve afferent fibers are formed early during embryonic development and link hair cells within the sensory epithelia of the different endorgans with 2°VN (see Straka, 2010). The somata of these afferents form the ganglion of Scarpa, which in amphibians is inconspicuously hidden in the VIIIth nerve at the level of its passage through the cranial wall (e.g., Dunn, 1978; Honrubia et al., 1989; Reichenberger and Dieringer, 1994; Straka et al., 1996). Bundles of afferent fibers from individual semicircular canal and otolith endorgans as well as their respective cell bodies in the ganglion are partially segregated with respect to their peripheral origin (Fritzsch et al., 2002). This tendency of separation into endorgan-specific fiber bundles is likely related to a spatially segregated topography and temporally dispersed formation of the different endorgans. With respect to various morpho-physiological properties such as resting discharge rate and regularity, afferent fibers of both larval (Gensberger et al., 2016) and adult frogs (Honrubia et al., 1989) form a broad spectrum. Despite the apparent continuum of distinguishing features, afferent fibers in *Xenopus* tadpoles segregate into two distinct categorical groups (Gensberger et al., 2015) with physiological characteristics, reminiscent of phasic and tonic 2°VN of adult frogs (Straka et al., 2004). The obvious duality of the vestibular afferent population in *Xenopus* larvae complies with a separation into frequency-tuned pathways as a major organizational principle of the VOR (Straka et al., 2009) and its implementation already early after hatching.

In larval *Xenopus* and adult ranid frogs, vestibular afferents terminate within a recipient area in the dorsal hindbrain between the cerebellar peduncle and the obex (Hellmann and Fritzsch, 1996; Birinyi et al., 2001; Straka et al., 2001; Chagnaud et al., 2015). After entering the brainstem, the bundle of afferent fibers splits into a rostral and a caudal branch that each ramifies extensively within a delimited area (Suarez et al., 1985; Straka et al., 2001). In addition, a noticeable number of vestibular afferents extend into the reticular formation at the level of the abducens nucleus and into the cerebellum. Tracing studies of the vestibular nerve also delineated a small, localized group of efferent neurons, with projections to all vestibular endorgans (Hellmann and Fritzsch, 1996; Straka et al., 2001, 2006; Chagnaud et al., 2015). These neurons, present in all vertebrates are located ventro-medially to the afferent termination area close to the facial branchiomotor nucleus.

**Figure 4:**
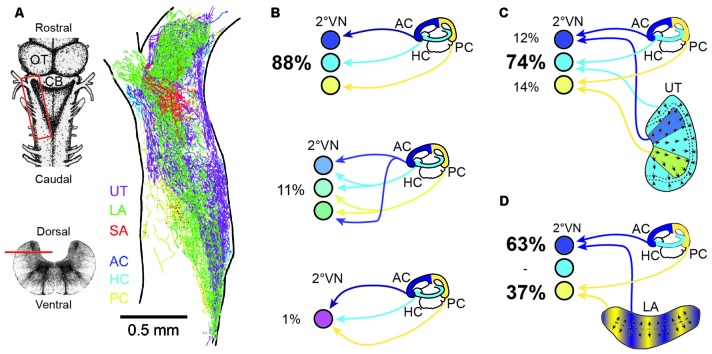
**Endorgan-specific afferent terminations in the vestibular nuclei and monosynaptic afferent semicircular canal and otolith signal convergence in frog 2°VN. (A)** Color-coded overlay of afferent terminal distributions from the three semicircular canals and the three otolith organs on a schematic horizontal section of an adult *ranid* frog; insets on the left show a dorsal view (upper scheme) and a cross-section of the brainstem (lower scheme) with the location of the section in **(A)** outlined by the red box and the horizontal bar, respectively. **(B)** Extent of convergence of monosynaptic inputs from one (top scheme), two (middle scheme) or all three (bottom scheme) ipsilateral semicircular canals in 2°VN. **(C,D)** Monosynaptic convergence between signals from the three semicircular canals and the utricle **(C)** or the lagena **(D)**; percentages in **(B,C)** indicate the relative proportion of 2°VN with monosynaptic responses from one or more semicircular canals **(B)** or from a semicircular canal and a particular otolith organ **(C,D)**. AC, anterior vertical semicircular canal; CB, Cerebellum; HC, horizontal semicircular canal; LA, lagena; OT, optic tectum; PC, posterior vertical semicircular canal; SA, saccule; UT, utricle. **(A–D)** Are based on data from Birinyi et al. (2001; **A**), Straka et al. (1997; **B**) and Straka et al. (2002b; **C,D**).

Despite the common placodal origin of the inner ear and lateral line mechanosensory systems in anamniotes, afferent fibers from the two locally separated hair cell sensory peripheries, occupy largely distinct, non-overlapping regions in the dorsal hindbrain (e.g., Chagnaud et al., 2015). This indicates the implementation of a topographical principle that is based on afferent projections into modality-specific, evolutionarily conserved central areas (Fritzsch et al., 2005). As shown in Axolotl, the developmental segregation of mechanosensory termination areas is a function of time and space. Later forming inner ear endorgans are innervated by later forming afferent connections that project centrally to more dorsal hindbrain areas (Fritzsch et al., 2005). However, while afferent projections from different mechanosensory systems are segregated, afferents from individual vestibular endorgans overlap to a large extent within the vestibular-recipient area of the hindbrain in amphibians as in other vertebrates (Figure 4A; Birinyi et al., 2001). This pattern, at variance with the well-established retinotectal map in *Xenopus* (e.g., Debski and Cline, 2002), indicates that the vestibular nuclei lack an obvious structured or layered topographic representation of the sensory periphery (Straka et al., 2000).

The rather negligible central afferent segregation suggests a considerable semicircular canal and otolith signal convergence at the level of individual 2°VN. In contrast with the highly overlapping termination areas of afferents from different inner ear endorgans (Figure 4A; Birinyi et al., 2001; Straka et al., 2003), adult frog 2°VN demonstrate a remarkably high selectivity for monosynaptic inputs from the sensory periphery (Straka et al., 1997, 2002b, 2003). As demonstrated in ranid frogs, most 2°VN select monosynaptic afferent inputs from only one semicircular canal (Figure 4B) and/or from only one otolith organ, despite the availability of inputs from all endorgans (Straka et al., 2002b). This indicates that the decomposition of angular and linear motion vectors by the spatial arrangement of the vestibular endorgans is largely maintained at the first neuronal level of the CNS. However, in many 2°VN, semicircular canal and otolith afferent inputs are combined monosynaptically in a specific manner (Figures 4C,D) that is related to the spatial orientation of the respective endorgans (Straka et al., 2002b). Accordingly, inputs from the horizontal otolith endorgan (utricle) combine preferentially with those from the HC in 2°VN (Figure 4C). In addition, afferent inputs from particular utricular sectors (see color-coded areas in Figure 4C) converge monosynaptically with signals from vertical semicircular canals, likely related to the co-stimulation of utricular and semicircular canal hair cells during tilt movements in the respective canal planes. In a complementary fashion, inputs from the vertical otolith organ (lagena in amphibians) converge in 2°VN exclusively with those from the anterior and PCs (Figure 4D). The ontogenetic establishment of this spatially specific convergence pattern likely requires an activity-dependent mechanism that allows a synaptic refinement based on co-activated sensory inputs during body motion in space. Accordingly, fine-tuning of the precise spatial specificity is likely based on a similar super-numerous production of exploratory axon collaterals as during the development of retinotectal pathways (Witte et al., 1996). Whether synapses from inappropriate connections are lost entirely or only silenced with the possibility of reactivation, as previously suggested after partial lesions of the VIII nerve, remains to be determined (Goto et al., 2000, 2002).

### Segmental Arrangement of Vestibular Projection

**Figure 5:**
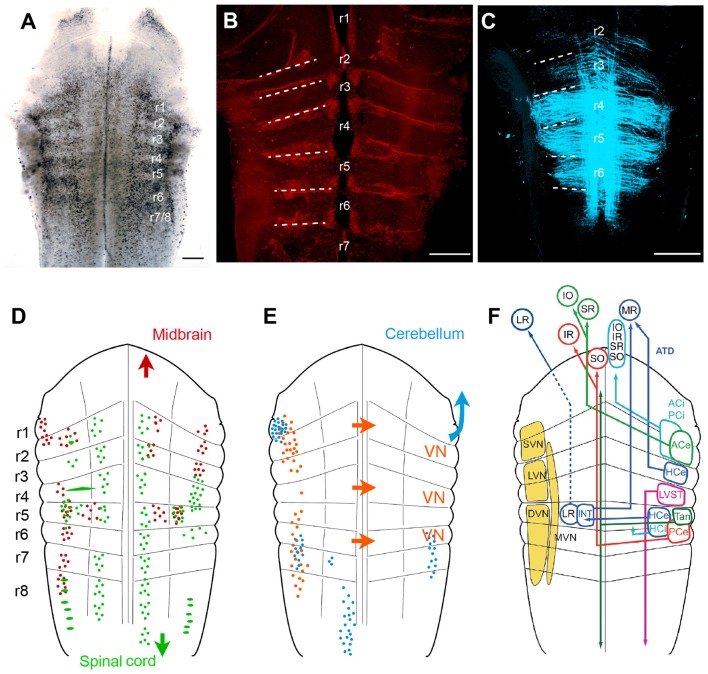
**Rhombomere-specific organization of vestibular projection neurons in larval frogs. (A–C)** Visualization of the rhombomeric scaffold by either naturally occurring segmentally iterated accumulation of dark pigments in the center of the rhombomeres **(A)**, by labeling with streptavidin Alexa 546, outlining the rhombomeric boundaries **(B)** or by long wavelength illumination (633 nm) outlining midline-crossing fibers within each rhombomere **(C)**; the material originates from brains of larval *Rana clamitans* (**A**; stage 26, according to Gosner, 1960) and *Xenopus laevis* (**B**, with permission from Hänzi et al., 2015a; **C**, with permission from Chagnaud et al., 2015); images in **(B,C)** were obtained from tadpoles at stage 52 according to Nieuwkoop and Faber (1994). **(D,E)** Schematics depicting the segmental arrangement of spinal-projecting (green in **D**), midbrain oculomotor nucleus-projecting (red in **D**), vestibular commissural (orange in **E**), and cerebellum-projecting neurons (blue in **E**). **(F)** Allocation of classical vestibular nucleus (VN) nomenclature (yellow) onto the larval amphibian rhombomeric scaffold and tentative segmental location of vestibular subgroups that mediate sensory signals from specific semicircular canals onto spatially matching extraocular and spinal motoneuronal populations. **(D–F)** adapted with permission from Straka et al. (2001). ACe, ACi, excitatory, inhibitory, anterior vertical semicircular canal neurons; ATD, ascending tract of Deiters; DVN, descending VN; HCe, HCi, excitatory, inhibitory horizontal semicircular canal neurons; INT, abducens internuclear neurons; IO, inferior oblique; IR, inferior rectus; LR, lateral rectus; LVN, lateral VN; LVST, lateral vestibulo-spinal tract; MR, medial rectus; MVM, medial VN; PCe, PCi, excitatory, inhibitory, posterior vertical semicircular canal neurons; r1–r8, rhombomeres 1–8; SO, superior oblique; SR, superior rectus; SVN, superior VN; Tan, tangential nucleus; Scale bars in **(A–C)** represent 250 μm.

Classical descriptions of central vestibular nuclei in the vertebrate hindbrain are based on anatomical features such as cell density, size and projection patterns (Figure 5; see Büttner-Ennever, 1992, 1999). These features allowed a separation into five subnuclei: a superior (SVN), medial (MVN), lateral (LVN) and descending vestibular nucleus (DVN) and a group Y. The entity of these nuclei forms the vestibular nuclear area and as such coincides with the brainstem recipient area for vestibular afferents (Figure 5F; Büttner-Ennever and Gerrits, 2004). This nomenclature is traditionally used in all vertebrates (see Straka and Dieringer, 2004; Straka et al., 2005) including amphibians (Matesz, 1979; Birinyi et al., 2001). However, the distinction into different subnuclei based on morphological features and hindbrain location prevents conceptualizing underlying organizational principles. More desirable would be a differentiation of vestibular neurons according to developmental origin or a compartmentalization into specific groups of functional phenotypes. An alternative reference frame that allows mapping spatial locations of defined subgroups of central vestibular neurons (Auclair et al., 1999; Cambronero and Puelles, 2000; Glover, 2000, 2003; Straka et al., 2001) is offered by the hindbrain segmentation (Vaage, 1969). While morphological visualization of the rhombomeric framework is usually restricted to vertebrate embryos, most species with a long larval development such as most amphibians retain a visible patterning (Figure 5A; Straka et al., 2002a). This scaffold therefore provides a link between hindbrain position of specific groups of vestibular neurons and their segmental origin, which governs the combinatorial expression of genetic regulatory elements (Gilland and Baker, 1993; Straka and Baker, 2013).

As a unique feature, hindbrain segments of larval frogs are often plainly visible as a rostro-caudal series of up to eight transversely oriented pigmented regions (Figure 5A; Straka et al., 1998, 2002a). However, segments can also be visually defined by glial cell accumulation at the boundaries (Yoshida and Colman, 2000), by labeling with streptavidin Alexa 546 (Figure 5B; Hänzi et al., 2015a) or simply by illumination with a wavelength between 610–650 nm (Figure 5C; Chagnaud et al., 2015). These segments correspond to the rhombomeric scaffold described in *Xenopus* embryos (Papalopulu et al., 1991). Independent of the visualization method, rhombomeres 2–6 (r2–6) appear as approximately similar-sized rostro-caudal brain regions (Figure 5A; see Straka et al., 2006). In contrast, r1 and r7/8 span across longer regions, respectively, and might in fact consist of multiple segments as suggested earlier (Gilland and Baker, 1993; Cambronero and Puelles, 2000). The visibility of the rhombomeric structure in amphibians, even at older larval stages or in adults, along with the absence of a post-embryonic longitudinal migration at least in anurans (Straka et al., 2006) facilitates a direct linkage between hindbrain location and ontogenetic origin of vestibular pathways (Straka, 2010).

#### Vestibulo-Ocular Neurons

Unilateral, spatially localized application of fluorescent tracers to the oculomotor and trochlear nuclei in larval anurans delineated an arrangement of backfilled vestibulo-ocular neurons at bilateral asymmetric hindbrain segmental positions. Backfilled neurons are located bilaterally in r1/2, ipsilateral in r3 and contralateral in r5–8, with the majority of neurons located in r5 and r6 (Figure 5D; Straka et al., 2001). Except for a few fibers, the main portion of crossed and uncrossed ascending axonal projections joins the contra- and ipsilateral medial longitudinal fascicle (MLF), respectively, to reach the trochlear and oculomotor nuclei. In addition, bilateral groups of abducens-projecting vestibular neurons occupy symmetric positions in r5 (Straka et al., 2001). The restricted location of abducens motoneurons in r5 in anurans (see below) indicates that the horizontal VOR circuitry is contained within a single hindbrain segment. Together with oculomotor- and trochlear-projecting vestibular neurons, the rostro-caudally distributed hindbrain positions comply with the location of homologous subgroups of VOR neurons in many vertebrate species (Suwa et al., 1996). Few segmental variations were found in bird and fish (Straka et al., 2014). The defined rhombomeric position and the reciprocal organization of excitatory and inhibitory VOR pathways (Precht, 1978; Straka and Dieringer, 2004) allow specifying the segmental origin of the plane-specific connections between semicircular canals and eye muscles (see color-coded arrangement in Figure 5F).

#### Vestibulo-Spinal Neurons

Spinal cord-projecting vestibular neurons in anurans are arranged in a segmentally specific pattern as revealed from tracing studies in *Rana* (Straka et al., 2001) and *Xenopus* larvae (Lambert et al., 2013). The most prominent cell group with uncrossed descending projections originates from r4 with minor contributions of neurons from the adjacent r3 and r5 segments. This group of vestibulo-spinal neurons corresponds to the large LVN neurons previously described in larval (Forehand and Farel, 1982; van Mier and ten Donkelaar, 1984) and adult anurans (ten Donkelaar et al., 1981). The major group of vestibulo-spinal neurons with crossed projections derives from a group of neurons in r5 with little contributions from adjacent segments (Figure 5D; Straka et al., 2001; Lambert et al., 2013). Descending axons of vestibulo-spinal neurons either join the MLF ipsi- or contralaterally or traverse laterally in a bundle that corresponds to the lateral vestibulo-spinal tract (LVST) described in birds and mammals (Glover, 2000). This cell cluster overlaps with contralateral oculomotor/trochlear nucleus-projecting vestibular neurons in r5, suggesting that this group of neurons with crossed, bidirectional projections complies with the graviception-related tangential nucleus of fish and birds (Díaz et al., 1998; Suwa et al., 1999; Straka and Baker, 2013). In contrast to mammals only very few neurons in this segmental location in anurans have bidirectional projections to oculomotor as well as spinal targets, indicating that vestibulo-ocular and vestibulo-spinal signals are processed in separate pathways (Straka et al., 2001). The major descending vestibular cell populations are supplemented by smaller groups of neurons with uncrossed spinal projections. These neurons are located medially to the tangential nucleus in r5 and more laterally in r6, with yet unknown equivalence to cell populations in other vertebrates (Straka et al., 2001). In addition, 1–3 particularly large vestibulo-spinal neurons with crossed projections are encountered in r2 along with a single Mauthner neuron in caudal r4 in all tadpoles and in all adults of permanently aquatic amphibian species (Cochran et al., 1980; Manns and Fritzsch, 1992; Straka et al., 2001; Lambert et al., 2013).

#### Vestibular Commissural Neurons

A major component of vestibular signal processing derives from the activity of brainstem commissural pathways that interconnect specific sets of neurons in the bilateral vestibular nuclei (Straka and Dieringer, 2004). In particular, the detection of angular head acceleration is centrally amplified by spatially specific inhibitory commissural pathways. These pathways interconnect, either directly or indirectly through additional local interneurons, central vestibular neurons with bilateral coplanar semicircular canal-related inputs (Holler and Straka, 2001; Malinvaud et al., 2010). In all studied amphibian species, the midline-crossing vestibular commissural neurons form two separate clusters of neurons (Figure 5D; Straka et al., 2001; Malinvaud et al., 2010). A rostral group of neurons is located in r1–3, with a predominance of cells in r2; the caudal group of neurons occupies r5–7, with the majority of neurons in r5 (Straka et al., 2001). Most notably, however, r4 is largely devoid of labeled neurons, visible as a gap between the two longitudinally extending subgroups of commissural neurons (Straka et al., 2001, 2002a). Commissural vestibular neurons are smaller than vestibulo-ocular or vestibulo-spinal neurons and consistently located medially to the latter two subgroups (Malinvaud et al., 2010). Axons of these commissural neurons cross the midline in the same segment in which the cell bodies are located with no additional axon collaterals to other targets. As shown in adult *ranid* frogs, the influence of the semicircular canal commissure on contralateral vestibular neurons is predominantly inhibitory (70%). These responses have mainly disynaptic onset latencies following electrical stimulation of individual semicircular canal nerves (Malinvaud et al., 2010). The semicircular canal plane-specific inhibition that is mediated directly by midline-crossing commissural fibers is GABAergic (Holler and Straka, 2001). This pharmacological profile is compatible with the presence of a substantial number of GABAergic commissural neurons with rostro-caudal hindbrain distributions that match the segmental origin of the two commissural subgroups (Malinvaud et al., 2010). Moreover, commissural inhibitory neurons selectively access tonic but not phasic 2°VNs, compatible with the exclusive possibility to modulate the discharge only in the former subtype (Beraneck et al., 2007; Malinvaud et al., 2010).

#### Vestibular-Related Precerebellar Neurons

The cerebellum of anuran tadpoles appears as a bilateral pair of small, dorso-medially directed laminae that are connected *via* the peduncles to the dorsal aspect of r1. The dorsal midline fusion is incomplete after hatching in anuran larvae and only finalized during larval development (Uray, 1985). The relatively late morphological completion of the cerebellar circuitry and interconnecting pathways complies with a delayed onset of a cerebellar control of vestibular reflexes (Gravot and Straka, unpublished observations). Determination of the origin of afferent connections at more advanced stages of amphibian tadpoles yield four main clusters of hindbrain neurons known to be involved in cerebellar-vestibular interactions (Figure 5E; Straka and Dieringer, 2004). The largest group of neurons forms an elongated nucleus ventro-medially in r8 on the contralateral side (Straka et al., 2001). The position and axonal trajectory complies with the location of inferior olivary neurons, which give rise to the climbing fiber input to the cerebellum in all vertebrates (Dieringer, 1974; Cochran and Hackett, 1977; Straka and Dieringer, 1992; see Llinás et al., 1969). Two smaller vestibular cell groups, located bilaterally in r6 and r7, along with direct vestibular nerve afferent projections give rise to mossy fiber inputs to the cerebellum and as such form part of the vestibulo-cerebellar feedback loop (see Llinás et al., 1969). The dense cell group lateral in r1 at the base of the cerebellar peduncle might be part of the cerebellar nucleus and thus another component of the feedback circuitry (Grover and Grüsser-Cornehls, 1984). The dense projection of fibers throughout the bilateral vestibular nuclei from r1 to r8 in larval and adult anurans after tracer injection into the cerebellum likely represent Purkinje cell terminals on vestibular neurons. This connection mediates a relatively weak (Magherini et al., 1975), nonetheless discernible and functionally relevant, GABAergic inhibition (Blanks and Precht, 1978; Pfanzelt et al., 2008).

### Organization of Extraocular Motoneurons and Eye Muscles

Extraocular motoneurons are located within the oculomotor, trochlear and abducens nucleus (Straka et al., 2014). Within the anuran oculomotor nucleus, situated immediately rostral to the midbrain-hindbrain boundary, individual eye muscle-specific populations (MR, SR, IR, IO motoneurons) are arranged in a partially segregated manner (Matesz and Székely, 1977). This spatial organization is reminiscent of the birth date-related pattern recently described for zebrafish oculomotor and trochlear motoneurons (Greaney et al., 2016). In contrast to the midbrain origin of oculomotor motoneurons, trochlear and abducens motoneurons in anurans originate from rostral r1 and from r5, respectively (Straka et al., 1998, 2006). The mono-segmental origin of the latter nucleus is similar to that reported for mammals but differs from the bi-segmental pattern of fish and birds, where abducens motoneurons derive from both r5 and r6 (see Straka et al., 2006; Gilland et al., 2014). In addition, abducens internuclear neurons with crossed ascending projections to contralateral MR motoneurons in the oculomotor nucleus are intermingled with abducens motoneurons in r5 (Straka and Dieringer, 1991; Straka et al., 2001). These neurons ensure an activation of bilaterally synergistic horizontal eye muscles in order to execute conjugate eye movements in anurans as in all other vertebrates (Straka and Dieringer, 1993). In a classical push-pull organizational scheme, all extraocular motoneurons receive reciprocal excitatory and inhibitory vestibular inputs from the bilateral vestibular nuclei that are spatially matched to the pulling direction of the eye muscles (see e.g., Figure 1C; Straka and Dieringer, 2004).

Eye muscles and extraocular motoneurons are generated early during amphibian embryogenesis and axonal bundles of all six groups of motoneurons appear morphologically complete and identifiable in *Xenopus* and Axolotl at the time of hatching (Sonntag and Fritzsch, 1987; Matesz, 1990a,b). However, the shape of the motoneuronal dendritic tree undergoes further post-embryonic maturation, visible as regionally restricted extension of the dendrites and an increase in branch number (Matesz, 1990a,b). If the earlier appearance of neuroblasts in the oculomotor compared to the trochlear and abducens nucleus is due to a rostro-caudal developmental gradient, or rather due to a methodological bias remains unclear (Matesz, 1990a,b). Even though extraocular motoneurons are formed by the time of hatching, their number increases during larval development in both anuran and urodeles (Sonntag and Fritzsch, 1987; Matesz, 1990a,b). The gradual augmentation of motoneurons parallels the concurrent increase in the number of extraocular muscle fibers in each eye muscle (Faust and Straka, unpublished observations). This suggests that the ratio for extraocular motor innervation is genetically determined and maintained into adulthood.

The early embryological establishment of eye muscles and motoneurons in amphibians as in other vertebrates (Glover, 2003; Straka, 2010) is compatible with the idea that the formation of the extraocular motor system and its wiring is one of the first steps in VOR circuit establishment and essential in creating the specific framework for mediating vestibular sensory signals onto spatially matching extraocular motor targets (Straka et al., 2014). The morphological completion of all structural elements and interconnections necessary for vestibular reflexes by the time of hatching, suggests that amphibian larvae have a fully functional VOR as soon as the tadpoles start to swim freely (Boothby and Roberts, 1992; Jamieson and Roberts, 2000). This notion fully complies with the interpretation derived from the different behavioral studies on VOR development in that the wiring between the vestibular sensory periphery and the extraocular motor plant has been established at the embryonic stage prior to locomotor onset. However, a further refinement and maturation occurs during subsequent larval stages.

## Conclusion

Amphibians provide excellent vertebrate systems to decipher ontogenetic steps and requirements for circuit assembly and the onset and maturation of gaze-stabilizing reflexes. Apart from few species-specific particularities, most results obtained so far in anurans and urodeles represent general characteristics of the vertebrate vestibular circuitry and its ontogeny. The easy accessibility of the amphibian VOR network at embryonic and larval stages has revealed particular aspects of the ontogenetic framework for establishing VOR functionality. The possibility to probe vestibular reflexes at behavioral and neural circuit levels gave insight into the constraints, which determine the onset of semicircular canal function and the contribution of the latter to the fine-tuning of the linear VOR. The use of amphibians for developmental analyses therefore provides unique opportunities to further advance our understanding of the requirements necessary for the assembly and maturation of the vertebrate gaze control system.

## Author Contributions

All authors have written the manuscript and created part of the figures. All authors listed, have made substantial, direct and intellectual contribution to the work, and approved it for publication.

## Conflict of Interest Statement

The authors declare that the research was conducted in the absence of any commercial or financial relationships that could be construed as a potential conflict of interest.
